# Stereodynamics Study of the Reaction of O(^3^P) with CH_4_ (v = 0, j = 0)

**DOI:** 10.3390/ijms10052146

**Published:** 2009-05-14

**Authors:** Yufang Liu, Yali Gao, Hongsheng Zhai, Deheng Shi, Jinfeng Sun

**Affiliations:** Department of Physics, Henan Normal University, Xinxiang 453007, China; E-Mails: hongsheng419@163.com (H.Z.); scattering@sina.com (D.S.); wlxyzfz@henannu.edu.cn (J.S.)

**Keywords:** quasiclassical trajectory, potential barrier, polarization, isotope effect

## Abstract

A new London-Eyring-Polanyi-Sato (LEPS) potential energy surface (PES) is used in the O + CH_4_ → OH + CH_3_ reaction via the quasiclassical trajectory method (QCT). Comparing with the experiments and the former *ab initio* calculations, the new LEPS PES describes the actual potential energy surface of the O + CH_4_ reaction successfully. The four polarization dependent “generalized” differential cross sections (PDDCS) are presented in the center of mass frame. In the meantime, the distribution of dihedral angle [*P*(*φ_r_*), the distribution of angle between *k* and *j*′ (*P*(*θ_r_*)] and the angular distribution of product rotational vectors in the form of polar plots in *θ_r_* and *φ_r_* (P(*θ_r_, φ_r_*) are calculated. The isotope effect for the reactions O + CD_4_ is also calculated. These results are in good agreement with the experiments.

## 1. Introduction

The reactions of ground-state atomic oxygen, O(^3^P), with hydrocarbons are the important initial steps of oxidation in combustion processes [1] and in Low Earth Orbit (LEO) conditions [2]. Among those reactions, the abstraction reaction O(^3^P) + CH_4_ → OH + CH_3_ has attracted considerable interest, both in experimental [3–7,11] and theoretical [8–10,12–20] calculations over the past decades. The rate constant values have been established in experiments [3,4]. Experimental studies have reported the translational energy release to the umbrella mode of the CH_3_ product [5] as well as OH ro-vibrational distributions [6,7]. The thermal rate constants have been measured with a wide variety of methods [8–10]. The state distribution of the OH product was found by experiments [5,6,8,11] and theoretical [12] calculations. A small amount of OH rotational excitation was found [5,6,11], which has been interpreted as resulting from a direct abstraction mechanism with a preferentially collinear O-H-C approach of the O(^3^P) atom attacking to the C-H bond. Theoretical calculations [8,9,12,13] showed that this reaction has a collinear O-H-CH_3_ transition state. The classical barrier height is around 0.4 eV [20,21]. Reduced-dimensionality quantum [16–19] models have been reported. The calculated kinetic behaviors and product state distributions are generally in good agreement with experimental findings [7].

It has been recognized that the correlated angular distribution provides an informative three-dimensional picture of a chemical reaction [22–25]. The angular distribution of the reagent and product relative velocity vector (*k*, *k*′) is characterized by the differential cross-section *dσ/dω_t_*. Furthermore, the angular distribution describing the relative orientation of vectors *k*, *k*′ and product rotational quantum number *j’* in space may be termed the *k - k’ - j’* distribution. The correlations among three vectors in the center-of-mass frame can be characterized by certain interesting double and triple vector correlations [26]. To our best knowledge, there is only one experimental work [27] relate to the product angular distribution (*k* - *k*′), scalar and two-vector properties of the reaction were analyzed using the QCT method [14], the full-dimensional trajectory [13,20] calculations only relate to the *k* - *k*′ angular distributions. So the full product angular distribution of this reaction has not been reported.

We calculated the product rotational polarization, the scattering-angle resolved product rotational alignment, the vector correlations of the reaction O(^3^P) + CH_4_ → OH + CH_3_ and the isotope effect for the reactions O + CH_4_/O + CD_4_.

## 2. Results and Discussion

**Figure 1.**
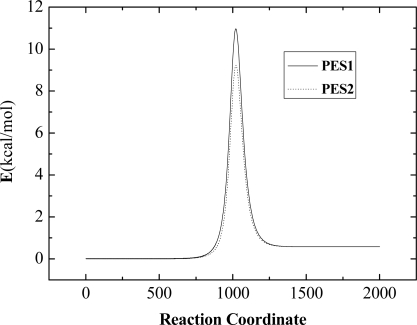
The reaction profile along the minimum energy paths of the O + CH_4_ reaction on the PES1 and PES2.

**Figure 2.**
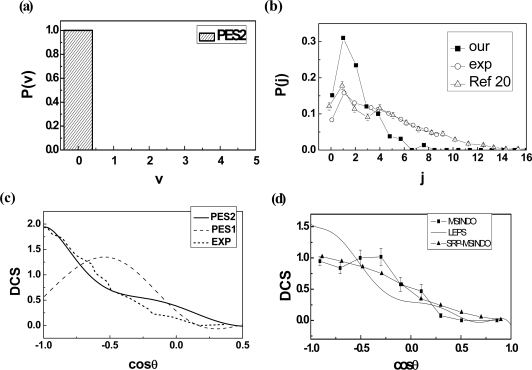
(a) The vibrational population of the product OH. (b) The rotational population of the products OH. The solid square symbol is our results, the circle symbol is experimental data, and the triangle symbol is taken from [20]. (c) The DCS distribution of the product OH at collision energy 0.54 eV. The solid line is the result on the PES2, the dash line is the result on the PES1 and the dot line is the experimental results. (d) The DCS distribution of the product OH at collision energy 0.65 eV. The solid line is our result, the solid line and solid square symbol are taken from [16], the solid line and solid triangle symbol are taken from [20].

Figure 1 shows the minimum energy paths of the reaction O + CH_4_ → OH + CH_3_ at the collision energy of 0.65 eV from reactants to products on our chosen PESs. A new LEPS PES with a different set of Sato parameters has been calculated in our work. The Sato C parameters from [33] are on the PES1 and the Sato D parameters which we calculated are on the PES2. We get the Sato D parameters from the *ab initio* calculation carried out by Troya and García-Molina [20] when the quality of the minimum energy path on the new LEPS potential energy surface accords with the experimental results. In Figure 1, there is a potential barrier in the reaction. The values of the potential barrier are 11 kcal/mol on PES1 and 9.23 kcal/mol on PES2. The results on PES2 are accord with the calculation [20] in which the barrier is around 9.22 kal/mol. The product’s ro-vibrational distributions are shown in Figure 2. From Figure 2(a) we can see that the most probable vibrational quantum number of OH is *v*^'^ = 0. In Figure 2(b) the most probable rotational quantum number of OH is *j*′ = 1, which is quite close to the calculation [20] and experiment [37], but the product OH of our calculation is colder than the experimental results. The reason is that the collision energy changes to the product’s translational energy rather than rotational energy in the O+CH_4_ direct abstraction reaction.

In the direct abstraction reaction, the rotational distribution is always in the narrow and low range of the rotational energy. The PDDCS (*2π/σ*)(*dσ_00_/dω_t_*), which is proportional to the differential cross section (DCS), predicts the angular distribution of the product molecular. In Figures 2(c,d) the DCS distribution is quite close to the experiment [27] and calculations [13,20]. The PES2 reflecting the real reaction process is much better than the PES1.

**Figure 3.**
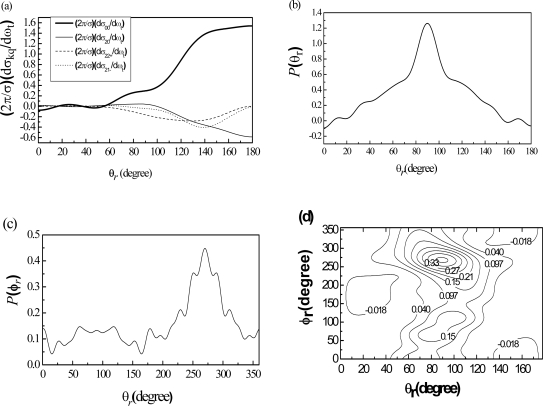
The collision energy is 0.65eV (a) Four PDDCS, boldfaced solid line indicating (*2π/σ*)(*dσ_00_/dω_t_*), thin solid line indicating (*2π/σ*)(*dσ_20_/dω_t_*), dash dot indicating (*2π/σ*)(*dσ_22_/dω_t_*) and short dot indicating (*2π/σ*)(*dσ_21-_/dω_t_*).(b) The distribution of *P*(*θ_r_*), reflecting the *k - J’* correlation.(c) The dihedral angle distribution of *J’*, *P*(*φ_r_*) with respect to the *k -k’* plane.(d) Polar plots of *P*(*θ_r_, φ_r_*) distribution averaged over all scattering angles.

The value of *(2π/σ)(dσ_20_/dω_t_)*, which is the expectation value of the second Legendre moment, shows the trend which is opposite to that of *(2π/σ)(dσ_00_/dω_t_)*. At the extremes of forward and backward scattering, the PDDCSs with q ≠ 0 are necessarily zero. At these limiting scattering angles, the *k -k’* scattering plane is not determined and the value of these PDDCSs with q ≠ 0 must be zero. The variations of the PDDCSs with k = 2 reflect changes in the rotational polarization with the scattering angles and suggest that the PDDCSs for the O+CH4 reaction contain important dynamical information. The four PDDCS of the OH product state are shown in Figure 3(a).

It indicates that the product OH scatters backward. The angular distribution that is asymmetric with *θ_r_* *= 90*° is characteristic of a direct reaction mechanism. The available energy is released as product translation energy rather than internal excitation, and the product internal excitation is quite cold. This is consistent with Figure 2.

The *P(θ_r_)* and *P(φ_r_)* distributions are shown in Figures 3(b,c). We can get better graphical representation of the products polarization for the reaction O + CH_4_. Figure 3(b) clearly shows that the distributions of the *k - j’* correlation P(*θ_r_*) peak at *θ_r_* angle close to *θ_r_* *= 90*° and is symmetric with *θ_r_* *= 90*°. It demonstrates that *j_OH_* is strongly aligned perpendicular to the line of centers. The distribution of the *k - k’ - j’* correlation *P(φ_r_)* is shown in Figure 3(c). The *P(φ_r_)* tends to be asymmetric about *φ_r_* *= 180*°, reflecting the strong polarization of angular momentum. There are two peaks of *P(φ_r_)*, respectively*φ_r_* *= 270*°and *φ_r_* *= 90*°. It implies that the angular momentum (*j_OH_*) of the most product molecules aligns along the CM y-axis. This behavior suggests that the reaction proceeds preferentially when the reactant velocity vector lies in the plane containing all three atoms. However, for an initially random orientation of reactant molecules the probability of such planar collisions is very low; thus, we can conclude that the given PES reorients or polarizes the plane containing the three atoms into the *k - k’* plane during the reaction process. The distribution of *P(θ_r_, φ_r_)* is presented in Figure 3(d); one peak appears at (*90°*, *270*°). This suggests that the OH products are preferentially polarized perpendicular to the *k - k’* plane. The *P(θ_r_, φ_r_)* distribution is not symmetric about *φ_r_=180*°, reflecting the nonzero values of the PDDCS *(2π/σ)(dσ_00_/dω_t_)* for the O + CH4 reaction. It is in good accordance with the distribution of *P(φ_r_)* that the dihedral angle distribution tends to be asymmetric with respect to the scattering plane.

**Figure 4.**
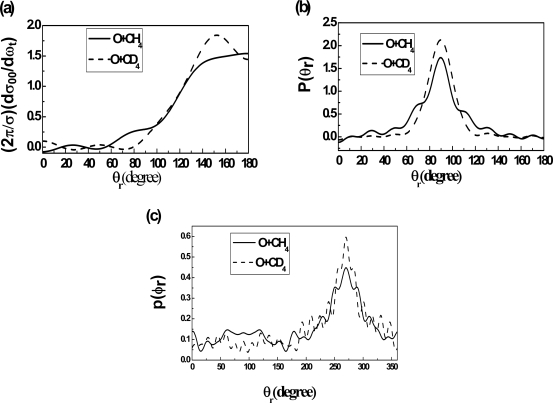
The collision energy is 0.65eV (a) The distribution of (*2π/σ*)(*dσ_00_/dω_t_*), reflecting the *k*−*k*′ correlation for O + CH_4_ and O + CD_4_ on the PES2. (b) The distribution of *P*(*θ_r_*), reflecting the *k - j’* correlation for O + CH_4_ and O + CD_4_ on the PES2. (c) The distribution of *P*(*φ_r_*), reflecting the *k - k’ - j’* correlation for O + CH_4_ and O + CD_4_ on the PES2. Solid line indicating the reaction O + CH_4,_ and short dot indicating the reaction O + CD_4_.

Figure 4(a) presents the PDDCSs *(2π/σ)(dσ_00_/dω_t_)* distributions of the products OH and OD at the collision energy of 0.65eV. The distribution of the products OD is a little more backward than that of the products OH. The increase of backward scattering with the mass number indicates that the rotational angular momenta distribution of the products is sensitive to the factor of merit [the factor of merit is cos^2^*β*=m_A_m_C_/(m_A_ + m_B_)(m_B_ + m_C_) for the reaction A + BC → AB + C] [32,34].

The product polarization distributions for the reaction O + CH_4_ and O + CD_4_ are shown in Figures 4(b,c) that describe the visible stereodynamics isotope effect. Figure 4(b) illustrates that the product distribution of *P(θ_r_)* for the O+ CD_4_ reaction is a little broader than that for O + CH_4_ reaction. This means that the rotational orientation effect of the product becomes weaker with the increase of the atomic mass. Han *et al.* [32,34]. have studied the product polarization for the reaction *H* + *H’*L (H, heavy; L, light), they found that the distribution of the product rotational angular momentum vectors is acutely sensitive to the mass factor, furthermore the increase of the mass factor can reduce the anisotropic distribution of the angular momentum *j*′ of the product molecule. The effect of mass factor cos^2^*β* = m_A_m_C_/(m_A_ + m_B_)(m_B_ + m_C_) on product rotational alignment is notable for the HHL mass combination reaction. The mass factor of the O + CD_4_ reaction is larger than that of the O + CH_4_ reaction. So the *j’* tend toward a less anisotropic distribution with respect to the vector k, while the rotation of the product from the O + CH_4_ is strongly aligned. During the reactive encounter, total angular momentum is conserved, *j* + *L* = *j’* + *L’* (here L and *L’* are the reagent and product orbital angular momenta). According to the impulse model and *j’* = *L*sin^2^*β* + *j*cos^2^β + *J*_1_m_B_/m_AB_, the larger product atom will take more angular momentum away, so the increase of the mass factor reduces the anisotropic distribution of *j*′.

Figure 4(c) describes that the *P(φ_r_)* distribution becomes a little broader when the H atoms are displaced by the D atoms. With the increase of the atomic mass, the preference for in-plane reaction c gradually hanges to a preference for an out-of-plane mechanism. The mass effect causes the reaction O + CD4 to prefer an out-of-plane mechanism. That is to say, it is not necessary that the product molecules rotate in the scattering plane containing all the three atoms when the reaction occurs. According to the [35] and [36], the *P*(*φ_r_*) is relevant to PES and the mass factor. We consider that the difference of the *P*(*φ_r_*) distribution is probably attributed to the different mass number and to the isotope effect. The obvious variation in the dihedral distribution implies that the mass effect plays an important role in the dynamical stereochemistry.

## 3. Experimental Section

### 3.1. Product rotational polarization in the center-of-mass (CM) frame

The center-of-mass (CM) frame is chosen; in this frame, the *z*-axis is parallel to the reagent relative velocity *k*, and the *y*-axis is perpendicular to the *xz*-plane which contains *k* and *k*′. The distribution of the angular momentum *j*′ of the product molecule is described by the function *f*(*θ_r_*), where *θ_r_* is the angle between *j’* and *k. f*(*θ*) can be represented by Legendre polynomial [26]:

(1) $$f(\theta_{r})=\sum a_{n}p_{n}(\text{cos}\;\;\theta_{r})$$

n = 2 indicates the product rotational alignment 〈*p*_2_(*j*′ · *k*)〉 = 〈3 cos^2^ *θ_r_* − 1〉/2, where *p*_2_ is the second Legendre moment, and the brackets indicate an average over the distribution of *j’* about *k*.

The full three-dimensional angular distribution associated with *k*, *k*′ and *j*′ can be represented by a set of generalized polarization-dependent differential cross-sections (PDDCSs) in the CM frame. The *k - k’ - j’* correlated CM angular distribution is written as the sum [26,29].

(2) $$p(\omega_{t},\omega_{r})=\sum_{kq}\frac{\left[k\right]}{4\pi }\frac{1}{\sigma }\frac{d\sigma_{kq}}{d\omega_{t}}C_{kq}(\theta_{r},\varphi_{r})*$$

where (*1/σ*)(*dσ_kq_/dω_t_*) is a generalized polarization-dependent differential cross-section (PDDCS), and (*1/σ*)(*dσ_kq_/dω_t_*) yields.

(3) $$\begin{array}{cc} \frac{1}{\sigma }\frac{d\sigma_{k0}}{d\omega_{t}}=0 & k\;\text{is}\;\text{odd}, \end{array}$$

(4) $$\frac{1}{\sigma }\frac{d\sigma_{kq+}}{d\omega_{t}}=\frac{1}{\sigma }\frac{d\sigma_{kq}}{d\omega_{t}}+\frac{1}{\sigma }\frac{d\sigma_{k-q}}{d\omega_{t}}=0,\;\;k\text{ is even},\;q\text{ is odd or }k\text{ is odd},\;q\text{ is even}.,$$

(5) $$\frac{1}{\sigma }\frac{d\sigma_{kq-}}{d\omega_{t}}=\frac{1}{\sigma }\frac{d\sigma_{kq}}{d\omega_{t}}-\frac{1}{\sigma }\frac{d\sigma_{k-q}}{d\omega_{t}}=0,\;\;k\text{ is even},\;q\text{ is odd or }k\text{ is odd},\;q\text{ is odd}.$$

The PDDCS is written in the following form:

(6) $$\frac{1}{\sigma }\frac{d\sigma_{kq\pm }}{d\omega_{t}}=\frac{1}{4\pi }\sum_{k_{1}}\left[k_{1}]S_{kq\pm }^{k_{1}}C_{k_{1}-q}(\theta_{t},0\right)$$

where the $S_{kq\pm }^{k_{1}}$ is evaluated using the expected value expression

(7) $$S_{kq\pm }^{k_{1}}=\langle C_{k_{1}q}(\theta_{t},0)C_{kq}(\theta_{r},0)[{\left(-1\right)}^{q}e^{iq\phi_{r}}\pm e^{-iq\phi_{r}}]\rangle$$

where the angular brackets represent an average over all angles.

The PDDCS with q=0 is presented by:

(9) $$\frac{1}{\sigma }\frac{d\sigma_{k0}}{d\omega_{t}}=\frac{1}{4\pi }\sum_{k_{1}}\left[k_{1}\right]S_{k0}^{k_{1}}p_{k_{1}}(\text{cos}\;\theta_{t})$$

where $S_{k0}^{k_{1}}$ is evaluated by the expected value expression:

(10) $$S_{k0}^{k_{1}}=\langle p_{k_{1}}(\text{cos}\theta_{t})p_{k}(\text{cos}\theta_{r})\rangle$$

The differential cross-section is given by:

(11) $$\frac{1}{\sigma }\frac{d\sigma_{00}}{d\omega_{t}}\equiv \;p(\omega_{t})=\frac{1}{4\pi }\sum_{k_{1}}\left[k_{1}]h_{0}^{k_{1}}(k_{1},0)p_{k_{1}}\;(\text{cos}\theta_{t}\right)$$

the bipolar moments $h_{0}^{k_{1}}(k_{1},0)$ are evaluated using the expectation values of the Legendre moments of the differential cross-section: $S_{00}^{k_{1}}=h_{0}^{k_{1}}(k_{1},0)=\langle p_{k}(\text{cos}\theta_{t})\rangle$.

In many photoinitiated bimolecular reaction experiments, we will be sensitive to only those polarization moments with *k* = 0 and *k* = 2. In order to compare calculations with experiments, (*2π/σ*)(*dσ_00_/dω_t_*), (*2π/σ*)(*dσ_20_/dω_t_*), (*2π/σ*)(*dσ_22+_/dω_t_*) and (*2π/σ*)(*dσ_21_/dω_t_*) are calculated. In the computation, PDDCSs are expanded up to *k*_1_=7, which is sufficient for good convergence.

The usual two vector correlations (*k - k’*, *k - j’*, *k’ - j’*) are expanded in a series of Legendre polynomials. The distribution of the *k - j’* correlation is characterized by *P*(*θ_r_*) and the *P*(*θ_r_*) can be written as [26–28]:

(12) $$p(\theta_{r})=\frac{1}{2}\sum_{k}\left[k]a_{0}^{k}p_{k}(\text{cos}\;\;\theta_{r}\right)$$

where the $a_{0}^{k}$ coefficients (polarization parameters) are given by $a_{0}^{k}=\langle P_{k}(\text{cos}\theta_{r})\rangle$ with the angular brackets stand for an average over all the reactive trajectories. In this paper, *p*(*θ_r_*) is expended up to *k*=18, which shows good convergence.

The dihedral angle distribution of the *k - k’ - j’* three-vector-correlation is characterized by angle *φ_r_* [28,30]. It has been shown that the distribution of dihedral angle *φ_r_* may be expanded as a Fourier series:

(13) $$p(\varphi_{r})=\frac{1}{2\pi }[1+\sum_{neven\;\geq 2}a_{n}\;\text{cos}(n\varphi_{r}\text{)}+\;\sum_{nodd\;\geq 1}b_{n}\;\text{sin}(n\varphi_{r})]$$

with *a_n_* = 2〈cos(*nφ_r_*)〉 and *b_n_* = 〈2 sin(*nφ_r_*)〉. In this computation, *p*(*φ_r_*) is expanded to n=24, which shows good convergence.

The joint probability density function of angles *θ_r_* and *φ_r_*, which define the direction of *j’*, can be written [31] as:

(14) $$p(\theta_{r},\varphi_{r})=\frac{1}{4\pi }\sum_{kq}[k]a_{q}^{k}C_{kq}{\left(\theta_{r},\varphi_{r}\right)}^{*}=\frac{1}{4\pi }\sum_{k}\sum_{q\geq 0}\left[a_{q\pm }^{k}\text{cos}(q\varphi_{r})-a_{q\mp }^{k}i\text{sin}(q\varphi_{r})]C_{kq}(\theta_{r},0\right)$$

The polarization parameter $a_{q}^{k}$ is evaluated as:

(15) $$\begin{array}{cc} a_{q\pm }^{k}=2\langle C_{k\left|q\right|}(\theta_{r},0)\text{cos}(q\varphi_{r})\rangle & \text{k}\;\text{is}\;\text{even}, \end{array}$$

(16) $$\begin{array}{cc} a_{q\mp }^{k}=2i\langle C_{k\left|q\right|}(\theta_{r},0)\text{sin}(q\varphi_{r})\rangle & \text{k}\;\text{is}\;\text{odd}\text{.} \end{array}$$

In the calculation, *p*(*θ_r_*,*φ_r_*) is expanded up to k=7, which is sufficient for good convergence.

### 3.2. Potential energy surface

The extended-London-Eyring-Polanyi-Sato (LEPS) potential energy surface (PES) is applied in our calculation [32]:

(17) $$U(R_{1},R_{2},R_{3},)=Q_{1}+Q_{2}+Q_{3}-{\left\{\frac{1}{2}[(J_{1}-J_{2})+(J_{2}-J_{3})+(J_{3}-J_{1})]\right\}}^{\overset{1}{\;}/\underset{2}{\;}}$$

where *Q_i_* =(*^1^E_i_* +*^3^E_i_*)/*2*, *J_i_* = (*^1^E_i_* −*^3^E_i_*)/*2*. *^1^E_i_* is defined as the diatomic Morse potential function, and *^3^E_i_* stands for the anti-Morse function:

(18) $${}^{1}E_{i}={}^{1}D_{i}\left({\left\{1-\text{exp}[-\beta_{i}(\gamma -\gamma_{0})]\right\}}^{2}-1\right),$$

(19) $${}^{3}E_{i}={}^{3}D_{i}\left({\left\{1+\text{exp}[-\beta_{i}(\gamma -\gamma_{0})]\right\}}^{2}-1\right).$$

where *^3^D_i_* *=* *^1^D_i_(1 - S_i_)/2(1 + S_i_)* and *S_i_* is an adjustable parameter. For the reaction of *A + BC → AB + C*, the subscript i = 1, 2, 3 indicate AB, BC and CA, respectively.

The experimental results and the *ab initio* calculations of the O + CH_4_ → OH + CH_3_ reaction show that the minimal barrier occurs at a collinear configuration. The alkyl radical does not possess significant internal excitation. So in this paper, the CH_4_ is treated as a H-CH_3_ pseudo-diatom for approximation. The classical Hamilton equations are integrated numerically for motion in three dimensions. Trajectories are initiated with the CH_4_ molecule in v=0 and j=0 levels and the collision energy is 0.65 eV. 100,000 trajectories are sampled, and the integration step size in the trajectories is chosen to be 0.1 fs for the stability of the calculation results. The parameters of extended-LEPS PESs are presented in Table 1 [33]. All the calculations performed here were done under adiabatic approximation.

## 4. Conclusions

We have presented a quasiclassical trajectory study of the product polarization from the O + CH_4_ → OH + CH_3_ reaction on the new LEPS PES by using a new Sato parameter. The differential cross section *(2π/σ)(dσ_kq_/dω_t_)* show that the scattering is predominantly in the backward hemisphere. For the vector correlation, the J_OH_ is aligned in the plane perpendicular to the line of center and the four PDDCSs give a good explanation about the vector correlation. From the isotope effect for the reactions O + CH_4_ and O + CD_4_ we find that there is a backward scattering tendency with the deuterium instead of hydrogen of the *(2π/σ)(dσ_00_/dω_t_)* distributions. And the angular momentum polarization (*P*(*θ_r_*) and *P*(*φ_r_*)) of OD is much stronger than that of OH.

## Figures and Tables

**Table 1.** The PES parameters for the O + CH_4_ → OH + CH_3_ reaction.

**Parameter**	**O-H**	**H-CH_3_**	**O-CH_3_**
*β_e_(*Å^−1^)^a^	2.294	1.83	1.96
*D_e_*(kJ/mol)^a^	445.34	447.26	384.35
*r*_e_^a^	0.9706	1.093	1.44
*Sato C*^a^	0.30	0.20	−0.15
*Sato D*^b^	0.70	0.3702	−0.4

^a^ Taken from Reference 33.

^b^ This work
